# A propósito del bicentenario de la independencia de Colombia: las prácticas de lectura de Antonio Nariño y el desarrollo de una vacuna presuntamente efectiva contra la viruela

**DOI:** 10.7705/biomedica.5024

**Published:** 2020-08-20

**Authors:** Sandra-Milena Moreno, Freddy Moreno

**Affiliations:** 1 Facultad de Ciencias de la Salud, Pontificia Universidad Javeriana, Cali, Colombia Pontificia Universidad Javeriana Facultad de Ciencias de la Salud Pontificia Universidad Javeriana Cali Colombia; 2 Facultad de Humanidades, Universidad del Valle, Cali, Colombia Universidad del Valle Facultad de Humanidades Universidad del Valle Cali Colombia

**Keywords:** viruela, vacuna contra viruela, historia, historiografía, Smallpox, smallpox vaccine, history, historiography

## Abstract

**Introducción.:**

La historia social de la cultura escrita reflexiona sobre los hábitos y prácticas que permiten apropiarse de los textos mediante la lectura y la escritura. De allí, que la biblioteca de un individuo permita comprender sus hábitos de lectura, su manera de imaginar la naturaleza, su relación con el poder político y religioso, y su vinculación con la sociedad.

**Objetivo.:**

Interpretar las prácticas de lectura de Antonio Nariño a partir de los libros de Medicina de su biblioteca, para aproximarse a la manera en que desarrolló una vacuna presuntamente efectiva contra la viruela.

**Materiales y métodos.:**

Se hizo una descripción bibliográfica de los documentos “Confiscación y embargo de bienes de Nariño” y “Papeles, libros y bienes de Sebastián López Ruiz en poder de Nariño” del Archivo Nariño de la Universidad Nacional de Colombia.

**Resultados.:**

De los 39 libros sobre Medicina (siete tratados de Cirugía, 12 compendios del ejercicio práctico, 11 manuales de enfermedades, siete compendios de temáticas médicas y dos libros sobre partos), tres eran tratados sobre la viruela.

**Conclusión.:**

El ejercicio médico-científico de Antonio Nariño refleja sus prácticas de lectura y de escritura, sus habilidades y sus competencias, y permite reconocer sus actitudes culturales y sociales ante la promoción de la noción de salud pública. El estudio de Nariño como médico autodidacta permitió relacionar las técnicas de producción científica (desarrollo de la vacuna) y la materialidad cultural (situación actual), a partir de los textos de Medicina de su biblioteca.

## Abstract (en)

**Introduction:**

The social history of written culture reflects the reading and writing habits and practices that allow us to appropriate the texts to build our sense of community. Hence, the library of individuals reflects their reading habits, their way of imagining nature, their relationship with political and religious power, and their involvement with society.

**Objective:**

To interpret Antonio Nariño’s reading practices by means of the medical books in his library to approach the way he developed a presumably effective vaccine against smallpox.

**Materials and methods:**

We made a bibliographic description of the documents “*Confiscación y embargo de bienes de Nariño*” and “*Papeles, libros y bienes de Sebastián López Ruiz en poder de Nariño*” from the Nariño Archive of the *Universidad Nacional de Colombia*.

**Results:**

Out of the 39 books about Medicine (seven treatises on surgery, 12 compendia of practical exercise, 11 disease manuals, seven compendia of medical topics, and two books on childbirth) three were smallpox treatises.

**Conclusion:**

Antonio Nariño’s medical and scientific practice reflects his reading and writing habits, his skills and competences, and his cultural attitudes, which promoted the notion of public health. The study of Nariño as a self-taught physician allowed for relating the scientific production techniques (development of the vaccine) and the cultural materiality (state of the art) based on the medical texts in his library.

La historia social de la cultura escrita, el estudio de la historia del libro, la historia de la edición o la historia de la cultura impresa, como procesos que se extienden a lo largo del tiempo, implican la reflexión sobre los diferentes modos, hábitos, prácticas y costumbres de lectura y de escritura, que permiten a las personas y a las instituciones apropiarse de la cultura del libro y sus derivados, ya sea para el fortalecimiento de una actividad u oficio (lectura utilitaria) o para el placer, por gusto o por recreación (lectura no utilitaria) ^1^.

Estos modos de utilización, comprensión y apropiación de los textos, así como su conservación y almacenamiento, dan origen a disposiciones que conducen a la construcción del sentido del mundo del lector y de las comunidades de interpretación. En dichas comunidades, los lectores comparten un conjunto de competencias íntimamente relacionadas con los textos (ideas), su materialización (libros) y las prácticas de lectura, las cuales se reconocen como las técnicas, los gestos y los modos de ser del lector en relación con la propia interpretación de dichos textos ^2^. En este sentido, las bibliotecas tienen un papel histórico fundamental, ya que cualquier actividad de lectura implica la existencia de un texto físico (manuscrito o impreso) que se preserva en un espacio físico, ya sea privado o público ^3^.

El estudio de la presencia y las funciones del libro impreso en la sociedad colonial neogranadina, ha permitido aproximarse a la circulación del libro en el ámbito de las asociaciones literarias urbanas, o tertulias, que a la manera de los espacios públicos modernos, favorecieron el desarrollo de una nueva forma de relacionarse con los textos por parte de los individuos de la élite letrada de la sociedad neogranadina ^4^. Así, el inventario de la biblioteca o librería de un individuo en particular puede dar pistas sobre sus hábitos y prácticas de lectura para dilucidar su forma de imaginar la naturaleza, de relacionarse con el poder político y religioso, y de vincularse con la sociedad ^5^. No obstante, el sentido y el alcance de los hábitos de lectura y de escritura obedecen a las condiciones sociales que permiten su compendio y comunicación, lo que demuestra que las prácticas y las representaciones son constitutivas de la realidad que, por ser simbólicas, son culturales ^6^.

En este contexto, tras la conformación del Virreinato de la Nueva Granada en 1718, se crearon numerosos y variados tipos de bibliotecas y librerías formadas por particulares, instituciones públicas y órdenes religiosas, cuyas colecciones permiten reconstruir la comunidad de lectores (quiénes y qué leían) que comenzó a formarse en el territorio americano durante la Colonia, a tal punto que su estudio en el marco de lo que se ha denominado ‘historia de la cultura impresa,’ ha posibilitado reconocer lo que se publicaba, lo que circulaba y las preocupaciones de las profesiones, toda vez que, de alguna manera, las bibliotecas y los libros que contenían dan testimonio de la formación de tales espacios públicos o privados y de su influencia cultural e ideológica ^7^^,^^8^.

Aunque los estudios referentes a las prácticas de lectura en la Nueva Granada son prácticamente inexistentes, sí hay reportes sobre la historia del libro como objeto material a partir de la historia de la imprenta, de las bibliotecas particulares, de los libros leídos por algunos personajes y de su comercio ^9^. Algunos de tales estudios historiográficos han precisado los inventarios parciales de las bibliotecas de los ilustrados neogranadinos a partir de actas de embargos, testamentos, recibos de compras, correspondencia y otros documentos. De esa forma, se han descrito las librerías del párroco Juan Fernández de Sotomayor ^9^, de José Celestino Mutis (principios del siglo XIX y 1786), de Juan José D’Elhuyar (1796), de Jorge Tadeo Lozano (1816), de Camilo Torres (1802) y de Antonio Nariño (1794) ^4^. De igual manera, se hizo con diferentes bibliotecas particulares: la del clérigo José Beltrán de Caicedo (1776), las de los burócratas José Francisco Martínez Bueno (1769), José Ignacio Paredo (1782) y Miguel de Escobar Ospina (1792), y las de los hacendados María Felipa de Rivas (1808) y Nicolás de Rentería (1821) ^8^.

De la lectura de los títulos de las colecciones, volúmenes y ejemplares, se ha obtenido invaluable información tanto de las nuevas búsquedas intelectuales de fines del siglo XVIII y de principios del siglo XIX, como del interés por la novedad editorial y la adopción de una nueva sensibilidad y de una actitud moderna frente a la cultura, lo que permite determinar una serie de transiciones, como el paso del latín al castellano y de los temas religiosos a los científicos (ciencias naturales, historia natural, medicina e higiene, nueva filosofía, economía política, periodismo y educación) ^8^; además, información sobre la motivación de la pequeña élite cultural que se estaba gestando en el Virreinato de la Nueva Granada para crear periódicos locales, fundar tertulias de lectura e intercambiar libros y gacetas ^10^. De esta forma, el mundo del libro, de la edición y de la impresión, abrió el camino a la divulgación de ideas y a su reproducción tipográfica para legitimar los procesos de independencia (últimas décadas del siglo XVIII) y la creación de un nuevo orden republicano (primeras décadas del siglo XIX) ^1^.

## El caso de Nariño

Don Antonio Nariño y Álvarez, hijo del contador de las cajas reales del Virreinato de la Nueva Granada, don Vicente Nariño y Vásquez y de Catalina Álvarez del Casal, nació en Santafé de Bogotá el 9 de abril de 1765. Adelantó cursos de gramática, filosofía, latín y griego en el Seminario Real Mayor de Santafé y en el entonces Colegio de San Carlos, regentado por la Compañía de Jesús. Posteriormente, fue reconocido como uno de los miembros más brillantes de la generación neogranadina, educado en el pensamiento ilustrado bajo la guía del médico y sabio científico José Celestino Mutis. Con él, Nariño formalizó su sentido humanista, fortaleció el manejo del francés, el italiano y el inglés, y se hizo experto científico en Medicina y en Botánica, a lo que debe sumarse su formación militar, su ejercicio como contador, tesorero y periodista, sus prácticas como librero e impresor, y sus conocimientos en política y economía. Sin embargo, su mayor reconocimiento se debe a la traducción y publicación (al parecer el 15 de diciembre de 1793) de los 17 artículos de la Declaración de los Derechos del Hombre y del Ciudadano, proclamada por la asamblea del pueblo francés el 4 de agosto de 1789, lo que le valió casi nueve años de encarcelamiento (había sido condenado a diez). Dicha declaración sería finalmente impresa y socializada por el mismo Nariño en 1811 ^11^.

La biblioteca de Nariño, heredada de su padre en 1778, contaba inicialmente con 108 títulos y 245 volúmenes, fundamentalmente de Derecho, Filosofía y Gramática, a los que se sumaron libros de otras disciplinas que, con el paso de los años, adquirió en virtud de su oficio de librero.

Como ya se mencionó, a raíz de la traducción, impresión y distribución de los ejemplares de la Declaración de los Derechos del Hombre y del Ciudadano, Nariño fue arrestado y sus bienes fueron confiscados por orden del oidor Joaquín de Mosquera y Figueroa en un proceso que comenzó el 29 de agosto de 1794 y finalizó el 3 de septiembre del mismo año. En el inventario de libros embargados en su casa de la Plazuela de San Francisco, el alguacil mayor de corte don José Gil Martínez Malo enumeró libros de los clásicos griegos, latinos, españoles y franceses, de política, filosofía y economía, de literatura, gramática y retórica, de jurisprudencia hispánica, historia clásica y crónicas de Indias, de medicina, botánica, física, química, geografía y mineralogía, de economía política, periodismo, educación y literatura y, por último, de devoción, apologética cristiana y teología, manteniendo las entradas de título, autor, tamaño del libro, tipo de encuadernación y número de tomos ^12^.

En cuanto a los libros de Medicina, la biblioteca de Nariño incluía textos de filosofía médica, fisiología, enfermedades, técnicas de cirugía y consejos prácticos de higiene, lo que evidencia su gusto de comerciante, burócrata y lector aficionado a los temas médicos ^4^^,^^13^. Presumiblemente, de ellos obtuvo la información necesaria para desarrollar una vacuna presuntamente efectiva contra la viruela.

## La viruela

Para dimensionar el trabajo de Antonio Nariño en la búsqueda de una vacuna contra la viruela, debe establecerse el contexto sobre la enfermedad y el virus que la producía. Cabe mencionar que, para la época de Nariño, la palabra ‘virus’ no se usaba para catalogar o clasificar la viruela, tal y como lo hacemos hoy en día. En el contexto de la microbiología, el término ‘virus,’ ‘veneno’ en latín, fue empleado por primera vez por Dmitri Iosifovich Ivanovski en 1892 para describir el virus del mosaico del tabaco, con lo que se inició el estudio formal de la virología ^14^.

Se especula que el virus causante de la viruela emergió en el año 10.000 a. C., pues se ha encontrado en restos momificados de la octava dinastía egipcia. Sin embargo, los primeros reportes datan del siglo IV en China y del siglo X en el suroeste de Asia. Dado que el virus no tiene reservorio animal y depende del paso entre humanos para sobrevivir, el rastreo ha evidenciado que salió de África a la India, pasó a Grecia y Roma, y se estableció en Europa. No sería hasta el siglo VI que el obispo Mario de Avenches le diera el nombre de ‘variola’ -del latín *varius* (mancha), y solo en el siglo XVIII se hizo el primer reporte epidemiológico de la enfermedad que registraba un número estimado de 400.000 muertes al año ^15^.

La viruela era ocasionada (en pasado, porque se considera erradicada desde 1980) por un virus perteneciente a la familia Poxviridae, la cual incluye los virus más grandes y complejos que pueden infectar a los seres humanos. Entre ellos están los del género *Orthopoxvirus* como el *Variola virus* (viruela humana), el de la viruela bovina, el de la viruela de los monos y el *Vaccinia virus,* que tienen forma ovoide y una morfología compleja, presentan una envoltura lipoproteica, un centro que contiene el ADN viral y dos cuerpos laterales que contienen proteínas virales, polimerasas y factores de transcripción ^16^^,^^17^.

*Variola virus* se clasifica en dos tipos, variola mayor y variola menor, con diferencias antigénicas mínimas, pero con manifestaciones clínicas que pueden variar de uno a otro, siendo más letal la variola mayor, con una mortalidad entre el 15 y el 40 %, en tanto que la variola menor tiene una mortalidad del 1 %.

Su transmisión ocurría por inhalación e infectaba inicialmente las células del epitelio respiratorio de las vías aéreas superiores, lo que producía una primera viremia, para después diseminarse a los ganglios linfáticos en donde se replicaba en las células fagocíticas; allí se producía una segunda viremia que se diseminaba a diferentes órganos (hígado, bazo, médula ósea y riñones) y a tejidos como la epidermis, en donde infectaba las células de las capas basal y media para generar necrosis y vesículas ^18^.

Las manifestaciones clínicas iniciales incluían malestar general, fiebre, mialgia, cefalea, vómito y diarrea y, posteriormente, aparecían las vesículas en la cavidad oral y en el resto del cuerpo. El exantema evolucionaba a partir de pápulo-vesículas que, aproximadamente, a los 12 días se convertían en pústulas que daban paso a costras. La muerte se presentaba por una toxemia caracterizada por coagulopatía, hipotensión arterial y falla orgánica múltiple ^19^.

Según algunos registros históricos, a finales del año 1.000 d. C. se desarrolló en la India una técnica de protección contra la viruela conocida como ‘variolización,’ la cual consistía en inocular a personas sanas a través de la piel o por inhalación nasal material extraído de las pústulas o costras de las lesiones de pacientes infectados. Esta técnica permitía que el paciente expuesto generara síntomas más leves que los asociados con la exposición natural. Si bien la técnica se extendió a China, Asia occidental y África, la enfermedad seguía teniendo una tasa alta de mortalidad, similar a la de la exposición natural.

Solo hasta 1796, Edward Jenner demostró que la inoculación en la piel de material extraído de la pústula de un humano infectado con viruela bovina generaba protección contra la infección natural por *V. virus*. Jenner llamó al material extraído ‘vacuna’ por proceder de la viruela que infecta al ganado vacuno, y llamó a la técnica ‘vacunación.’ Al comienzo, la vacunación se hizo con la técnica de inoculación ‘brazo a brazo’ y mediante el empleo de ‘hilos impregnados’ con el contenido de las pústulas, los cuales también se empleaban para transportar el virus a grandes distancias, aunque en ocasiones perdía la viabilidad y la vacunación no era exitosa. La evidencia del éxito de la vacunación era la aparición de lesiones pustulares que evolucionaban hasta una costra ^15^^,^^19^.

Ya en el siglo XX, la vacuna de la viruela consistía en virus vivos atenuados de la cepa *Vaccinia virus*, cuyo origen se desconoce ^20^, aunque algunos autores han señalado que podría estar relacionado con una ‘quimerización’ ocurrida durante la experimentación con los virus de la viruela humana y la bovina, en tanto que otros autores han sugerido un origen equino ^16^^,^^17^.

Esparza, *et al.*, han señalado que, desde la creación de la vacuna, Jenner pensó que podía existir una combinación de virus equinos y vacunos, y en su publicación “La consulta” proponía la hipótesis de que la viruela bovina se había originado en los caballos y había sido transmitida a las vacas por los granjeros. Además, cuando se creó la vacuna y se usó la técnica de inoculación ‘brazo a brazo,’ pudo haber ocurrido la recombinación genética entre las cepas vacunas y las equinas ^21^.

De esta forma, el virus inoculado induce la activación de los linfocitos B que secretan anticuerpos protectores, así como células TCD8+ citotóxicas y células Th1 que secretan citocinas, lo que favorece la reacción inmunológica que contrarresta la viremia. Los anticuerpos y las células de memoria pueden tener una duración aproximada de 30 años ^20^.

En el caso de Nariño, el objetivo fue analizar sus prácticas de lectura con base en el rastreo de los libros de Medicina que se encontraron en su biblioteca particular, para aproximarse a la posible manera en que desarrolló una vacuna presuntamente efectiva contra la viruela. En este sentido, se hizo una descripción bibliográfica analítica del documento 53 del tomo I de la “Confiscación y embargo de bienes de Nariño” y del documento 86 del tomo II de los “Papeles, libros y bienes de Sebastián López Ruiz en poder de Nariño”, disponibles en el Archivo Nariño, con el propósito de exponer las características de los libros que conformaban su biblioteca, específicamente de aquellos cuyo título y descripción temática hicieran alusión a la Medicina y que pudieran dar cuenta de la manera como Nariño adquirió habilidades y competencias teórico-prácticas para llevar a cabo un ejercicio experimental como el requerido para el desarrollo de una vacuna contra la viruela.

El Archivo Nariño, disponible en la biblioteca digital de la Universidad Nacional de Colombia (http://www.bdigital.unal.edu.co/8059/), constituye la mayor y más completa obra documental publicada hasta ahora sobre el “precursor de la independencia”, producto de más de 50 años de investigación de Guillermo Hernández de Alba, quien llevó a cabo la búsqueda, el cotejo y la verificación de las fuentes. El archivo comprende el período de los años de vida de Antonio Nariño (1765-1823), además de algunos documentos anteriores a su nacimiento y posteriores a su muerte, los cuales han sido clasificados en seis tomos: tomo I (infancia y juventud, 1727-1795), tomo II (infancia y juventud, 1795-1810), tomo III (presidencia de Cundinamarca, 1809- 1812), tomo IV (presidencia de Cundinamarca, 1812-1814), tomo V (campaña del sur, 1812-1815) y tomo VI (cárcel, destierro y regreso, 1816-1823) ^22^.

En el documento sobre la “Confiscación y embargo de bienes de Nariño” (cuadro 1), entre otros, se relacionan 21 libros cuyos títulos aluden a temas de Medicina y en los cuales se identifica el autor y se señala la cantidad de tomos, la empastadura, el tipo de papel y el tamaño. Las temáticas médicas incluyen tres tratados de cirugía, 12 compendios del ejercicio práctico de la medicina y seis manuales de enfermedades, en dos de los cuales se describen las inoculaciones para tratar la viruela. Uno de estos libros corresponde a la disertación de Francisco Gil impresa en 1784, en la que se discute la legitimidad de prevenir la viruela inoculando la enfermedad a una persona sana con muestras de otra persona enferma ^23^. El otro libro, cuyo autor no se menciona en el inventario de confiscación, podría ser la “Disertación sobre la inoculación de las viruelas” de Bonifacio Jiménez de Lorite, publicada en 1758, o la de Francisco Rubio, impresa en 1769 ^24^. Ruiz ha señalado que podría tratarse de un segundo ejemplar de la obra de Francisco Gil ^13^.

**Cuadro 1:** Listado de libros sobre temas de Medicina confiscados a Antonio Nariño en 1794

Título	Autor	Descripción	Año
Cirugía	Elie Col de Vilars	Un tomo, en pasta, en octavo	1746
Diccionario de cirugía	Sue Lejdunne	Dos tomos, en pasta, en cuarto	1777
Fundamentos de la materia médica	Federico Cartheuser	Un tomo, en pasta, en octavo	1755
Compendio de toda la medicina práctica	Laurencio Heister	Tres tomos, en pasta, en octavo	1776
La salud del hombre	Benito Arias Montano	Un tomo, en pasta, en cuarto	1571
Medicina	Armando Bourdon	Un tomo, en pasta, en octavo	1776
General sistema de la cirugía	Laurence Heister	Un tomo, en pasta, en cuarto	1719
Practis Medica	Lazare Riviére	Un tomo, en pasta, en cuarto	1651
Observaciones acerca de las enfermedades	John Pringlue	Dos tomos, en pergamino, en cuarto	1755
del ejército en los campos y las guarniciones			
Medicina	Amato Lusitano	Un tomo, en pergamino, en cuarto	1588
Disertación físico-médica en la cual se	Francisco Gil	Un tomo, a la rústica, en cuarto	1784
prescribe un método seguro para preservar			
a los pueblos de viruelas hasta lograr la			
completa extinción de ellas en todo el reino			
Medicina doméstica	Buchán	Un tomo, en pasta, en cuarto	1784
Medicina	Amado Bourdon	Seis tomos, en pasta, en octavo	1776
Tratado de las enfermedades de las mujeres	Jean Astrug	Cinco tomos, en pasta, en octavo	1785
Medicina	Guillermo Buchan	Segundo y quinto tomos, en pergamino, en octavo	1785
Nuevas utilidades de la quina	José Aslet	Un tomo, en pasta, en octavo	1774
Elementos de medicina	Guillermo Cullen	Tres tomos, en pasta, en octavo	1791
Observaciones generales sobre los hospitales	Liberti Fromondi	Un tomo forrado en papel, en pergamino, en cuarto	1788
Disertación sobre la inoculación de las viruelas	No se reporta	Un tomo, en pergamino, en cuarto	-
Medicina	Richard Morton	Un tomo, en pasta, en cuarto	1689
Disertación medicinal	Servatio Auguftino de Villers	Un tomo, en pergamino, en octavo	1748

Fuente: documento 53 del tomo I del Archivo Nariño*

* El orden de los libros obedece a la secuencia en que iban siendo confiscados e inventariados durante los cuatro días de la diligencia de las diferentes habitaciones de la casa de Nariño ubicada en la Plazuela de San Francisco.

En el documento “Papeles, libros y bienes de Sebastián López Ruiz en poder de Nariño” (cuadro 2), se encontraron 18 libros de Medicina de los cuales se descartaron cinco porque, siendo de López, hicieron parte del inventario de confiscación. Estos incluían tomos de cuatro tratados de cirugía, siete compendios de temáticas médicas generales, dos libros sobre partos y cinco manuales sobre enfermedades, de los cuales uno era una disertación sobre la viruela traducida por Juan Espallarosa e impresa en 1767, en la cual se demostraba la utilidad y la seguridad de la inoculación de las viruelas ^24^. Según las dos fuentes y la revisión que hizo Ruiz ^13^ de la biblioteca del prócer, Nariño tuvo contacto, por lo menos, con tres libros sobre viruela.

**Cuadro 2:** Listado de libros sobre temas de Medicina de Sebastián López Ruiz que estaban en poder de Nariño

Título	Autor	Descripción	Año
Compendio de medicina	No se reporta.	Siete tomos, en pasta, en octavo	-
Enfermedades venéreas y de la uretra	Thomas Goulard	Dos tomos, en pasta, en octavo	-
Señales de muerte actual	Brugier	Dos tomos, en octavo	-
Memorias de cirugía	No se reporta.	Dos tomos, en pasta, en octavo	-
Enfermedades venéreas	Edme Claude Bourru	Un tomo, en pergamino, en octavo	1786
Partos	Cardin Le Bret	Cuatro tomos, en pasta, en cuarto	-
Cirugía expurgada	Johannes de Gorte	Un tomo, en pergamino, en cuarto	1780
Cirujano instruido	Thomas Goulard	Un tomo, en pergamino, en cuarto	1774
Aforismos de cirugía castellana	Wansurieten	Cinco tomos, en pergamino, en cuarto	-
Embriología sagrada	Francesco Cangiamila	Dos tomos, en pergamino, en cuarto	1785
Farmacopea matritense	Real Colegio de Boticarios de Madrid	Un tomo, en pergamino, en cuarto	1739
Materia médica	No se reporta.	Cinco tomos, en pergamino, en cuarto	-
Hipócrates	No se reporta.	Un tomo, en pergamino, en folio	-
Diccionario anatómico	No se reporta.	Dos tomos, en pergamino, en octavo	-
Tratado de las enfermedades de las mujeres	Joseph Raulin	Un tomo, en pergamino, en octavo	1783
paridas, con el método de curarlas			
Morbis oculorum	Herman Boerhaave	Un tomo, en pasta, en octavo	1748
Disertación físico-médica, en que con la	Juan Espallaroza	Un tomo, en pasta, en cuarto	1767
razón, autoridad y experiencia se demuestra			
la utilidad y seguridad de la inoculación de las			
viruelas			
Diccionario botánico y farmacéutico	No se reporta.	Un tomo, en pasta, en octavo	-

Fuente: documento 86 del tomo II del Archivo Nariño*

* El orden de los libros obedece a la secuencia en que fueron presentados en el expediente instaurado en 1797 por Sebastián López Ruiz sobre unos papeles, libros y bienes que dice Antonio Nariño recibió de María Begoña Aldana.

Según Silva ^4^^,^^7^, a Antonio Nariño le fueron incautados entre 630 y 710 libros (distribuidos en 1.803 a 1.881 volúmenes, con algunos ejemplares repetidos), cantidad que revela su gran afición a la lectura y su oficio de comerciante de libros, así como su actividad de préstamo de libros. Para Ruiz ^13^, el número de libros incautados fue de 700 títulos (1.874 volúmenes) según el documento de la diligencia de confiscación. De estos, el 10,4 % (74 ejemplares) correspondía a libros de ciencias naturales, medicina y matemáticas, área temática que ocupaba el tercer lugar en cantidad, lo que evidencia los intereses y las preferencias de Nariño como lector, propietario, librero y vendedor ^4^. Si bien Ruiz ^13^ encontró 20 libros de medicina en el inventario del embargo, en el presente estudio se contaron 39 libros de Medicina, 21 de ellos en el inventario de confiscación. La diferencia radica en que, en nuestro estudio, se incluyó el libro “Nuevas utilidades de la quina”, debido al uso y aplicación de esta planta en Medicina.

Además de estos títulos, en diferentes archivos históricos se ha podido comprobar que varios miembros de la élite ilustrada neogranadina, entre ellos Jerónimo Torres, Pedro Fermín Vargas y el mismo Antonio Nariño, poseían conocimientos médicos adquiridos mediante la lectura que les permitían actuar, no solo como mediadores o intermediarios culturales en la transmisión de conocimientos de diferentes aspectos de la salud al resto de la población, sino también, en la prestación de servicios de atención, recetario y formulación médica debido a la escasez de médicos titulados en la Nueva Granada ^10^.

A partir de los manuales de salud, estos neogranadinos pudieron obtener información sobre ‘la medicina de los pobres,’ en la que se recurría al uso tradicional de plantas medicinales para tratar a los enfermos ^25^.

Nariño, por su parte, también conoció obras fundamentales como el “Avis au peuple sur sa santé” de Samuel André Tissot, tal como lo sugiere la correspondencia de Mutis referenciada por Sotomayor ^26^. Este tratado, publicado en 1761 y considerado el texto fundador de la medicina social, se empleó para justificar el establecimiento de medidas de salud pública en la Nueva Granada. Debido a que los capítulos 13 y 23 están consagrados a la viruela y a su inoculación, respectivamente, este libro resultó primordial para las autoridades virreinales en el momento de enfrentar las epidemias de viruela que azotaron el territorio de la actual Colombia en 1782, 1783, 1785 y 1801, además de ser la base referencial de los principales escritos de Mutis sobre la viruela: “Sobre las precauciones que deben observarse en la práctica de la inoculación de las viruelas” de 1783 y “Método general para curar las viruelas” de 1802 ^26^.

## Desarrollo de la vacuna contra la viruela

La viruela, enfermedad infectocontagiosa causada por *V. virus,* que no tenía tratamiento específico y se manejada de forma preventiva mediante técnicas de variolización y vacunación, llegó a América a principios del siglo XVI en los barcos que transportaban a los esclavos desde África. En el “Almanaque Peruano” y en la “Gaceta de México” de 1801, se encuentran noticias sobre las gestiones para introducir en América la vacuna y la vacunación desarrolladas por Jenner y que consistían en infiltrar en el cuerpo humano la linfa de una pústula obtenida de otro humano contagiado por contacto con la ubre de una vaca (*cowpox*), método que estaba siendo usado con éxito en Europa ^27^.

En la Nueva Granada, las técnicas de variolización llegaron a finales del siglo XVIII promovidas por Mutis, quien estudiaba y difundía el mecanismo para prevenir y menguar los efectos de los brotes epidémicos de viruela que se daban en varias regiones del virreinato. Si bien la inoculación del material varioloso debía ser realizada por médicos, su carencia obligó a que religiosos, curanderos, parteras, boticarios y barberos efectuaran las incisiones requeridas para el inóculo como medida preventiva.

Se calcula que durante el brote de 1785, fallecieron 3.000 de las 15.000 personas que habitaban Santa Fe de Bogotá; sin embargo, en el brote de 1802, por el esfuerzo de haber mantenido la variolización, la cantidad de personas fallecidas no superó las 300 ^28^^,^^29^. Hasta ese entonces, el tratamiento preventivo consistía en la inoculación o paso del material varioloso de unos niños a otros, a través de hilos de algodón retorcido empapados en la “materia de las viruelas”, o de costras que se introducían por la nariz o por la introducción del material varioloso mediante incisiones con una lanceta ^30^:

Solo ante la epidemia de 1802 y dada la ineficacia de la variolización, tras consultar con el Consejo de Indias, el rey Carlos IV decretó la vacunación obligatoria por Real Cédula del 25 de abril de 1805 en todo el reino y aprobó la Real Expedición de la Vacuna al mando del médico Francisco Balmis, quien años atrás había traducido al castellano la obra “Tratado histórico y práctico de la vacuna” de Jacques Moreau de la Sarthe ^31^.

Por correspondencia con el virrey Mendinueta, Mutis era conocedor del método profiláctico de la vacuna descubierta por Jenner y, sirviéndose de su sobrino Sinforoso Mutis, inclusive, había recibido una vacuna proveniente de España. No obstante, llegó inactiva y no pudo usarse, por lo que el gobierno se vio obligado a recomendar nuevamente la variolización durante la epidemia de 1801 a 1802 ^25^. El médico José Salvani fue el segundo al mando de la Real Expedición de la Vacuna, quien llegó a Santafé de Bogotá el 17 de diciembre de 1804 con dos niños portadores de la infección para realizar más de 2.000 vacunaciones y establecer la junta provisional para la conservación de la vacuna encargada de las jornadas de vacunación y de preservar el fluido vacuno de Jenner en su forma activa ^26^^,^^30^^,^^32^.

Dado que la Nueva Granada fue la más afectada por la viruela, un neogranadino ilustrado fue el que más se interesó por el desarrollo de la vacuna en Europa. Antonio Nariño, aun estando encarcelado, llevó a cabo una práctica experimental que le permitió obtener una ‘vacuna’ presuntamente efectiva contra la viruela, la cual ensayó en su sobrino José María Ortega Nariño en el verano de 1802, un año antes de la llegada de la Real Expedición con la vacuna de Jenner. Nariño escribió una carta al virrey Mendinueta el 30 de julio del mismo año (documento 94 del tomo II, “Carta de Nariño al virrey Mendinueta” del Archivo Nariño), en la que narra sus experimentos:

“[…] Penetrado de los mismos sentimientos que han animado al superior gobierno desde que se tuvo noticia positiva de los laudables efectos de la vacuna, he procurado hacer cuantas tentativas me permite la estrechez de mi situación; y después de 47 días de trabajo, en que me han salido infructuosas varias experiencias, tengo hoy la satisfacción de presentar a vuestra excelencia un muchacho, en quien ha prendido un grano con todas las apariencias de verdadera vacuna, habiéndose seguido todos los períodos y síntomas que prescriben las dos únicas recetas que han llegado a esta capital, estando hoy precisamente en el día nono de la vacunación” ^22^.

Nariño era conocedor de la vacuna de Jenner y del procedimiento de vacunación, como lo demuestra su empleo del término en la carta. Además, su ejercicio empírico demuestra que, empleando técnicas de variolización, pudo inocular a un individuo que podía ser fuente de futuras vacunas, tal y como iban a ser utilizados los niños que venían con la Real Expedición de la Vacuna ^31^.

Aunque Mutis había manifestado que la variolización de las viruelas era una operación tan sencilla que cualquier persona podía practicarla con facilidad, la elección de los sujetos, la diversidad de preparaciones, el tiempo y las consideraciones de casos en los que no era posible la inoculación del material varioloso (mujeres en embarazo, niños en edad de recambio dental, etc.), exigían ciertos conocimientos reservados a la inspección del médico ^32^, conocimientos que Nariño posiblemente aprendió de la lectura de los libros de Medicina de su biblioteca, de la continua asesoría de los médicos Luis Francisco de Rieux y Manuel Antonio Froes, con quienes atendía y recetaba a los enfermos más pobres, de las tertulias con los médicos padre Miguel Isla y don Pedro Fermín Vargas, y de las constantes visitas que le hacían en su sitio de reclusión los médicos Honorato Villa y Sebastián López Ruiz, quienes, además del mismo Mutis, cuidaban de su precario estado de salud dadas las deficientes condiciones de su confinamiento en una celda contigua a los servicios comunes del cuartel de caballería de Santafé. Muy seguramente, fueron estos tres médicos los que le proporcionaron a Nariño los instrumentos e insumos necesarios para desarrollar la vacuna ^10^.

En mayo de 1803, luego de ser excarcelado bajo fianza por el oidor don Juan Hernández de Alba tras leer el informe médico de Mutis, Nariño se trasladó a la finca de su familia en Fucha para recuperarse ^33^.

En conclusión, siempre se ha hecho referencia a Antonio Nariño como militar, abogado y contador, prócer y precursor de la emancipación de las provincias americanas del imperio español, dejando de lado el hecho de que, en su calidad de practicante empírico de la Medicina, pudo ser una de las primeras personas que desarrolló una vacunación presuntamente efectiva con el propósito de contar con una vacuna contra la viruela en territorio americano.

Poco o nada se ha escrito sobre las condiciones de encarcelamiento de Nariño y la manera como estableció las dinámicas particulares de estudio y trabajo científico de este proceso más allá de las visitas de sus médicos para cuidar de su salud en una cárcel donde no tenía acceso a ningún libro o manuscrito de consulta, ya que su biblioteca, formada en las primeras tres décadas de su formación autodidacta, se encontraba completamente desarticulada, pues algunos ejemplares habían sido subastados y, los que no, terminaron en los repositorios de la Biblioteca Real para reposar, hoy en día, en la Biblioteca Nacional de Colombia.

Con base en elementos metodológicos de la historia cultural y a partir del estudio de las prácticas de lectura y escritura de Nariño, se pudo hacer una aproximación al significado de su ejercicio médico-científico y a su importancia para la salud pública:

[…] Espero que vuestra excelencia, siguiendo sus benéficas miras, lo mandará reconocer por los facultativos, y que de cualquier modo que resulten los efectos, no mirará en este paso sino un testimonio de mi amor al bien público y de mis vivos deseos de cooperar en todo con las intenciones del gobierno, únicos motivos porque me he ocupado en un objeto que tanto interesa en las actuales circunstancias a la salud pública […]” ^22^.

Sin embargo, no ha sido posible establecer si el sobrino de Nariño se consideró como potencial fuente de obtención de la vacuna, si esta fue probada en otros pacientes o si se usó en las jornadas de vacunación a partir de 1804 junto con la de Jenner.

Con base en la contextualización de la epidemia de viruela que azotó la Nueva Granada entre 1801 y 1804, se logró establecer la relación entre la viruela y las actitudes de los ilustrados neogranadinos frente a la enfermedad en el marco de los esfuerzos de la variolización de Mutis, y la llegada y distribución por todo el territorio de la vacuna de Jenner a cargo de la Real Expedición de la Vacuna.

De todas maneras, se destaca el trabajo efectuado por Nariño, quien a pesar de estar encarcelado y enfermo, logró desarrollar una vacuna presuntamente efectiva contra la viruela utilizando sus habilidades y competencias, y atendiendo a sus actitudes culturales y sociales fincadas en una mentalidad patriótica tradicional y en una concepción moderna determinada por el pensamiento ilustrado, según lo dejan traslucir sus hábitos y prácticas de lectura orientados hacia una noción de salud pública basada en el interés de la comunidad.

La intención de desarrollar una vacuna contra la viruela fue un reflejo evidente del pensamiento ilustrado neogranadino y, aunque no se tenga evidencia de su uso, en este estudio se pudo evidenciar que, atendiendo a los postulados de la historia social de la escritura y la lectura y del análisis de las prácticas y representaciones, esta fue producto del esfuerzo de Nariño a partir de estrategias materiales y de apropiación del conocimiento.

El estudio de Antonio Nariño como médico autodidacta permitió interpretar la relación entre las técnicas de producción científica (desarrollo de la vacuna) y la materialidad cultural (situación actual) a partir de los textos impresos de Medicina disponibles en la Nueva Granada entre finales del siglo XVIII e inicios del siglo XIX. En este sentido, los conceptos de la llamada ‘historia social de la cultura escrita’ han permitido relacionar un conjunto de prácticas que, desde diferentes posturas teóricas y metodológicas, se aproximan a las temáticas asociadas con esas prácticas, aunque muchas de las dinámicas y estructuras sociales permanecen todavía inexploradas ^34^.
